# Quality Evaluation of *Oviductus Ranae* Based on PUFAs Using HPLC Fingerprint Techniques Combined with Chemometric Methods

**DOI:** 10.3390/foods8080322

**Published:** 2019-08-07

**Authors:** Hongye Guo, Yuanshuai Gan, Min Liu, Shihan Wang, Shuling Ni, Yan Zhou, Yao Xiao, Zhihan Wang, Yongsheng Wang

**Affiliations:** 1 School of Pharmaceutical Sciences, Jilin University, Changchun 130021, Jilin, China; 2 College of Chinese Medicine Materials, Jilin Agricultural University, Changchun 130118, Jilin, China; 3 Department of Physical Sciences, Eastern New Mexico University, Portales, NM 88130, USA

**Keywords:** *Oviductus Ranae*, PUFAs, HPLC, fingerprint, chemometrics

## Abstract

*Oviductus Ranae* is a nutritional product for both medicine and food. Its quality is uneven due to multiple factors. An efficient method was established to evaluate the quality of *Oviductus Ranae* using fingerprint techniques and chemometric methods based on the analysis of polyunsaturated fatty acids (PUFAs) in petroleum ether extract by high performance liquid chromatography (HPLC). During this process, 27 batches of *Oviductus Ranae* were analyzed by HPLC and two types of chromatographic fingerprints were established. The fingerprint that was obtained by matching six known peaks was used for the quantification of six PUFAs. Another fingerprint was obtained by matching sixteen peaks with a peak area ratio greater than 0.5% and it was used to classify the different qualities of *Oviductus Ranae* by further combining three different chemometric models. The 27 batches of *Oviductus Ranae* were divided into four categories, which was consistent with the analysis results of six PUFAs contents. The two high-quality samples with significantly higher contents were classified into one category, and samples with medium contents were divided into two categories, including eight and thirteen samples, respectively. The four inferior samples with lower contents were classified into one category. The results indicated that the newly developed method has potential application prospects for the quality evaluation of *Oviductus Ranae*.

## 1. Introduction

**Figure 1:**
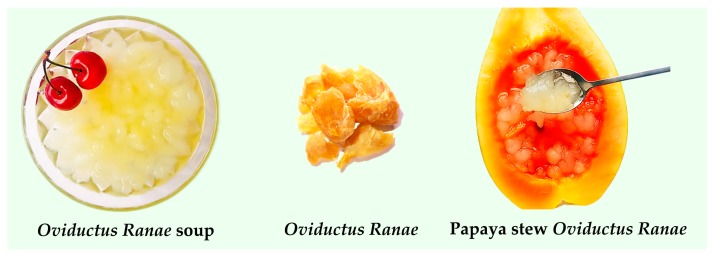
*Oviductus Ranae* sample and its typical edible methods, including *Oviductus Ranae* soup and Papaya stew *Oviductus Ranae*.

*Oviductus Ranae*, which is the dried oviduct of female *Rana temporaria chensinensis* David, is mainly produced in the Changbai Mountains area of Jilin Province, China [1,2,3,4]. *Oviductus Ranae* contains a variety of nutrients, such as polyunsaturated fatty acids (PUFAs), protein, and vitamin, so it is often regarded as a tonic to improve health [5,6,7]. Researches have shown that *Oviductus Ranae* has significant effects in enhancing immunity, anti-fatigue, anti-aging, and lowering blood fat [2,3,4,8,9,10], and it has been applied to various nutrient foods (Figure 1) [11,12]. *Oviductus Ranae* is usually collected from multiple regions as a nutritional product for both medicine and food. The quality of *Oviductus Ranae* from different sources is uneven due to the influence of various factors, such as climatic factors, breeding environment, harvesting techniques, and storage conditions [13,14]. Therefore, it is essential to establish an effective method for evaluating the quality of *Oviductus Ranae*.

The quality of nutritional product is closely related to the bioactive substances [15,16,17,18]. Through the study of different solvent extracts (ethanol extract, petroleum ether extract, and water extract) of *Oviductus Ranae*, it was found that the petroleum ether extract of *Oviductus Ranae* has relatively superior pharmacological activity [13,19], especially in terms of anti-depression [20]. Further identification of the ingredients revealed that the petroleum ether extract of *Oviductus Ranae* is rich in PUFA compounds, including the members of omega-3 fatty acid family (eicosapentaenoic acid (EPA), α-linolenic acid (ALA), and Docosahexaenoic Acid (DHA)), the members of omega-6 fatty acid family (arachidonic acid (ARA), linoleic acid (LA), and oleic acid (OA)), and other unsaturated fatty acid compounds [20,21,22]. PUFAs play important physiological functions in organisms [23,24]. Omega-3 fatty acids can reduce the risk of cardiovascular diseases, stimulate brain development, and they reduce the incidence of depression [25,26]. In addition to reducing the risk of metabolic syndrome, Omega-6 fatty acids also have the function of preventing cardiovascular disease [27,28]. Therefore, the PUFAs are potential indicators for the quality evaluation of *Oviductus Ranae*.

Chromatographic fingerprint with multiple variables is considered to be a comprehensive qualitative and quantitative analysis method [29,30], and the World Health Organization (WHO) also accepts the use of chromatographic fingerprint as a strategy for the identification and quality assessment [31]. Chromatographic fingerprints are complex multivariate data sets due to the complexity of samples, so minor differences among very similar chromatograms are still important [32]. Therefore, chemometric pattern recognition methods, such as hierarchical clustering analysis (HCA), principal component analysis (PCA), and partial least squares linear analysis (PLS-DA), are considered for the evaluation to reasonably distinguish the quality differences of the samples [33,34]. In fact, fingerprint combined with chemometric resolution methods has been widely used in the analysis of various types of foods [35]. For example, Devi et al. applied the method to identify the botanical origin of unifloral and multifloral honeys [36]. Chen et al. analyzed the quality of *Ganoderma lucidum* from different producing areas [32]. Garrido-Delgado et al. applied the method to determine the origin, quality and adulteration of the olive oil [37]. All of those examples showed the reliability of this method.

In this work, we analyzed the PUFAs in the petroleum ether extract of *Oviductus Ranae* by high performance liquid chromatography (HPLC) and then established a fast and reliable quality evaluation method of *Oviductus Ranae* by further using fingerprint techniques and chemometrics. Firstly, 27 batches of *Oviductus Ranae* samples were collected from the main producing areas of Changbai Mountains in Jilin Province, China, and the fingerprints of *Oviductus Ranae* were established. Afterward, we evaluated sixteen common peaks and six high content PUFAs (EPA, ALA, DHA, ARA, LA, and OA) in the fingerprint. Finally, different quality *Oviductus Ranae* samples were classified while using chemometric methods, including HCA, PCA, and PLS-DA.

## 2. Materials and Methods

### 2.1. Chemicals and Samples

Petroleum ether, methanol, and phosphoric acid were analytical pure and purchased from Beijing Chemical Factory (Beijing, China). HPLC-grade acetonitrile and HPLC-grade methanol were purchased from Fisher (Fisher Scientific, Fair Lawn, NJ, USA). The ultrapure water was prepared from a gradient water purification system (Water Purifier, Sichuan, China). Eicosapentaenoic acid (EPA, Lot No: 32510020), docosahexaenoic acid (DHA, Lot No: 0501808), and arachidonic acid (ARA, Lot No: 2491801) were purchased from TanMo Quality Testing Technology Co., Ltd. (Beijing, China). α-Linolenic acid (ALA, Lot No: U-62A-J25-C), linoleic acid (LA, Lot No: U-52A-JA3-C), and oleic acid (OA, Lot No: U-46A-MA6-C) were purchased from ANPEL Laboratory Technologies (Shanghai) Inc. (Shanghai, China). The purity of each standard compound was higher than 99%, as determined by HPLC. Six kinds of PUFAs standards were formulated to different concentrations using HPLC-grade methanol and filtrated with a 0.22 μm filter membrane prior HPLC injection.

**Figure 2:**
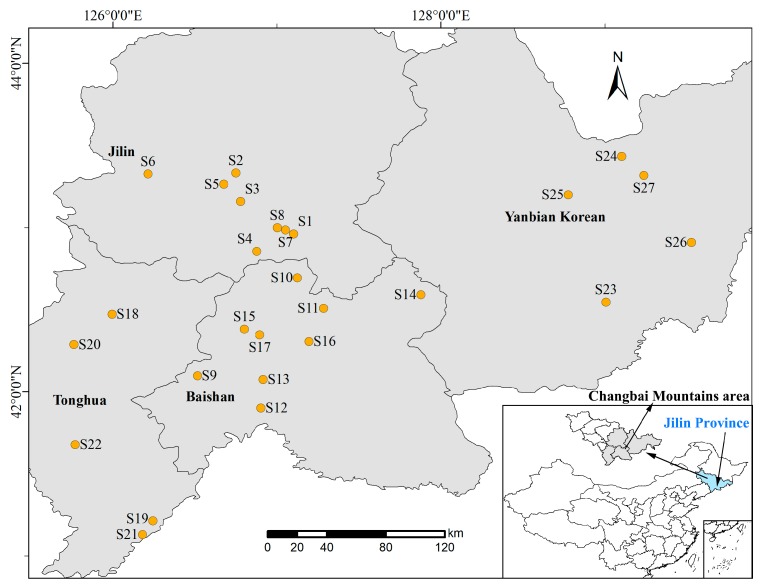
Regional distribution of 27 batches of *Oviductus Ranae* in Changbai Mountains area.

Jilin Province Rana Industry Association provided a total of 27 samples of *Oviductus Ranae*, which were collected from the Changbai Mountains area in Jilin Province, China. Collection sources covered the major production areas of Changbai Mountains. The specific collection information is shown in Table 1, and Figure 2 shows the geographical distribution of the samples.

### 2.2. Fatty Acid Extraction

Each *Oviductus Ranae* sample was pulverized into powders (passing through a 20-mesh sieve). Three copies of 0.80 g powders were weighed in parallel for each sample, wrapped with filter paper, and then placed in Soxhlet extractors. 70 mL petroleum ether was added to a 100 mL round bottom flask. The extraction device was placed in a constant temperature water bath at 90 °C for 6 h [21,38]. After the extraction was completed, the petroleum ether was spun dry under reduced pressure at 45 °C while using a rotary evaporator. Finally, the extract was dissolved with methanol and made up to volume in a 2 mL volumetric flask. The solution was filtered through a 0.22 μm filter before HPLC detection.

### 2.3. HPLC Chromatography Analysis

The HPLC analysis was performed on an Agilent Technologies 1260 Series liquid chromatograph (Agilent Technologies, Pittsburgh, PA, USA) that was equipped with a quaternary pump, autosampler, thermostatic column chamber, and ultraviolet (UV) detector. Chromatographic separation was achieved on an Agilent TC-C18 column (250 × 4.6 mm, 5 μm). The mobile phase consisted of HPLC-grade acetonitrile (A) and 1% aqueous phosphoric acid (B) while using a gradient program of 86–93% A in 0–10 min., 93% A in 10–20 min, 93–100% A in 20–30 min and a gradient flow rate of 1.0–0.5 mL/min in 0–14 min., 0.5 mL/min in 14–30 min. The detection wavelength was set at 203 nm, the column temperature was 30 °C, and the injection volume was 10 μL. The standards and samples were determined under the same HPLC conditions. The data were recorded and processed while using Agilent Chemstation (Agilent Technologies, Pittsburgh, PA, USA) software.

### 2.4. Validation of the Method

The method’s precision was determined by injecting the same solution six times in the same day while using the same chromatographic conditions. Six separate solutions of the same sample (sample S1) were analyzed to assess the repeatability. The stability of the sample solution was evaluated by analyzing the same sample solutions stored at room temperature for 0, 2, 4, 8, 16, and 24 h.

Three representative samples (S26, S22, and S12 representing low, medium, and high PUFA contents, respectively) were taken quantities of 0.80, 1.60, and 3.20 g, and were extracted in three replicates to validate the extraction process.

### 2.5. Establishment of Chromatographic Fingerprint

The common peaks and similarities of chromatographic fingerprint were analyzed while using the Similarity Evaluation System for the Chromatographic Fingerprint (2012 edition, Beijing, China) according to the suggestions of China Food and Drug Administration (CFDA). The chromatographic fingerprint data of all samples were imported into the system. The time window was set to 0.2 s and the calibration method was multi-point calibration. Through matching the common peaks of the markers, the reference chromatogram fingerprint was generated by using the average method. The similarity of chromatographic fingerprint data is indicated by the correlation coefficient (similarity). The closer the correlation coefficient is to 1, the higher the similarity between the samples. In this study, the correlation coefficients of all chromatograms of 27 batches of *Oviductus Ranae* samples were calculated and analyzed.

### 2.6. Data Analysis

In this study, all of the samples were weighed in triplicate for extraction and HPLC analysis. The relative standard deviation (RSD) of three repetitions of the same sample was always less than 5%. The peak area of the sixteen common chromatographic peaks in the sample was the average of three determinations. Before chemometric analysis, we normalized the area of each chromatographic peak of 27 *Oviductus Ranae* samples and performed unit variance scaling to meet the requirements of analysis. The chemometric analysis was performed while using the common chromatographic peaks as variables (*n* = 16) and the *Oviductus Ranae* samples as cases (*n* = 27), yielding a matrix of 432 data points.

#### 2.6.1. Hierarchical Clustering Analysis (HCA)

HCA is a multivariate analysis method that simplifies complex data through data modeling [39]. The results of the analysis are drawn into tree-like pedigrees, which could clearly show the classification results of the samples and facilitate the visual analysis of the similarities and differences among samples [40]. In this study, the samples of *Oviductus Ranae* were grouped by SPSS (version 25.0, SPSS Inc., Chicago, IL, USA), based on the clustering method of inter-group connections and the Squared Euclidean Distance interval.

#### 2.6.2. Principal component analysis (PCA)

As a statistical method of dimension reduction, PCA decomposes the covariance matrix to obtain the principal components (i.e., eigenvectors) of the data and their weights (i.e., eigenvalues), thereby recombining the original variables into a new set of independent comprehensive variables [41]. According to the actual situation, extracting several eigenvectors that are in front of the eigenvalues reflect the main information of the original variables, which can simplify the complex problems [40,42]. In this work, PCA was performed while using SPSS (version 25.0, SPSS Inc., Chicago, IL, USA), and the fractional scatter plot was interpreted by the relationship between PC1 and PC2 for visual analysis of the data matrix.

#### 2.6.3. Partial Least Squares Discrimination Analysis (PLS-DA)

PLS-DA is a multivariate analysis method with supervised statistical models [43]. It is a regression extension of PCA [41,44], which maximizes the variance covariance between classes by priori classification of given objects and it minimizes the variance covariance within classes under simultaneous consideration of all the analyzed features [45]. In the PLS-DA model, the parameters R^2^X(cum), R^2^Y(cum) are calculated by the cross-validation procedure to evaluate the goodness of fit, and Q^2^(cum) is used to describe the validity of the model. The values of these three parameters are between 0 and 1, the closer to 1 the more reliable the model [46,47]. In our work, the data were analyzed by PLS-DA to construct the analysis model and for classifying *Oviductus Ranae*. The program was processed on SIMCA (version 14.1, MKS Umetrics, Malmö, Sweden).

## 3. Results and Discussion

### 3.1. PUFA Standards Analysis

**Figure 3:**
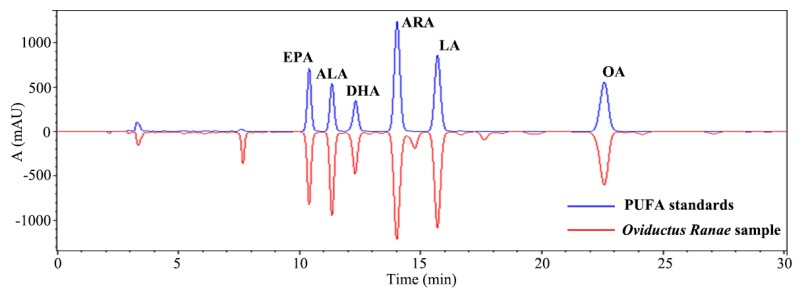
Comparison of PUFAs mixed standard with petroleum ether extract of *Oviductus Ranae.* Eicosapentaenoic acid (EPA), α-linolenic acid (ALA), Docosahexaenoic Acid (DHA), arachidonic acid (ARA), linoleic acid (LA), oleic acid (OA), polyunsaturated fatty acid (PUFA).

According to the literatures, the petroleum ether extract of *Oviductus Ranae* is rich in PUFA compounds [20,22]. Therefore, the mixed standard solution of PUFAs (composed of EPA, ALA, DHA, ARA, LA, and OA) and the petroleum ether extract of *Oviductus Ranae* (sample number S1) were analyzed while using the developed HPLC method. The chromatogram was shown in Figure 3. The six kinds of PUFAs in the standard solution and the sample solution all achieved good separation effect (minimum separation degree > 1.54), and the retention time of the PUFAs in the sample solution was determined.

The standard curves of six standards were used for the quantitative analysis of six kinds of PUFAs in *Oviductus Ranae* samples. The regression equations of six components were calculated in the form of standard curve (y = ax + b), where X and Y were the concentration and peak area, respectively. Table 2 shows the results of the regression equations. All of the standard curves showed good linearity in the test range (*R^2^* > 0.9982).

### 3.2. Methodology Validation

The feasibility of separating and analyzing six kinds of PUFAs in *Oviductus Ranae* samples by HPLC was validated with the RSD. The RSD results of retention time and peak area of six kinds of PUFAs are listed in Table 3, respectively.

In the results of the precision experiment, the values of RSD for the retention time and peak area were below than 0.65% and 3.01%, respectively. The RSD of the retention time and peak area in the repetitive results was less than 1.86% and 3.62%, respectively. The stability results showed that the RSD of the sample retention time and peak area within 24 h were less than 1.35% and 0.92%, respectively. All of the results showed that the method met the requirements of PUFA fingerprint analysis in *Oviductus Ranae*.

The feasibility of Soxhlet extraction of PUFAs from *Oviductus Ranae* was verified by determining the contents of PUFAs (μg/g) in three representative samples of different weights. The contents of PUFAs (μg/g) in sample S26 with low content of 0.80, 1.60, and 3.20 g were similar, and the RSD values were less than 5.42%. The difference of the PUFA contents (μg/g) in the medium-content sample S22 and the high-content sample S12 of different weights was slight. The RSD values were less than 4.08% and 5.55%, respectively. The above results showed that the extraction method was feasible. The specific data were showed in the Supplementary Materials Table S1.

### 3.3. Fingerprint Analysis

**Figure 4:**
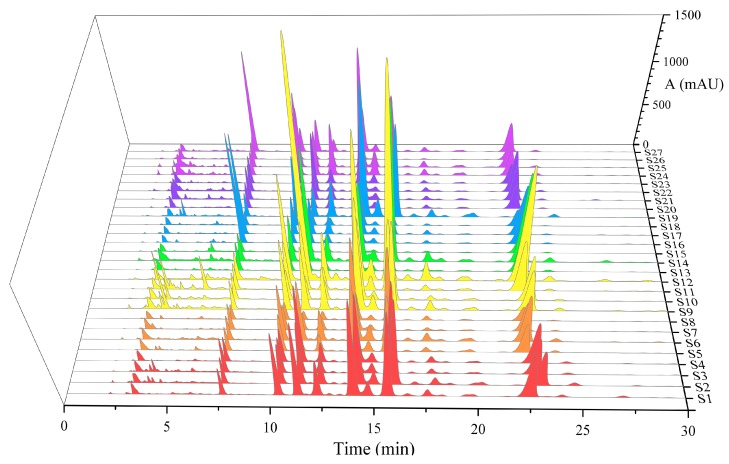
High performance liquid chromatography (HPLC) chromatogram of 27 batches of *Oviductus Ranae.*

The consistency of chemical composition is an important factor in the evaluation of the quality of traditional Chinese medicine. Therefore, a variety of PUFA components in *Oviductus Ranae* were investigated by chromatographic fingerprint. *Oviductus Ranae* from 27 sampling sites in the Changbai Mountains was analyzed by HPLC. Their chromatograms all had similar profiles, as shown in Figure 4. In the Similarity Evaluation System for the Chromatographic Fingerprint (2012 edition, Beijing, China), two different forms of chromatographic fingerprints were established by two matching methods.

**Figure 5:**
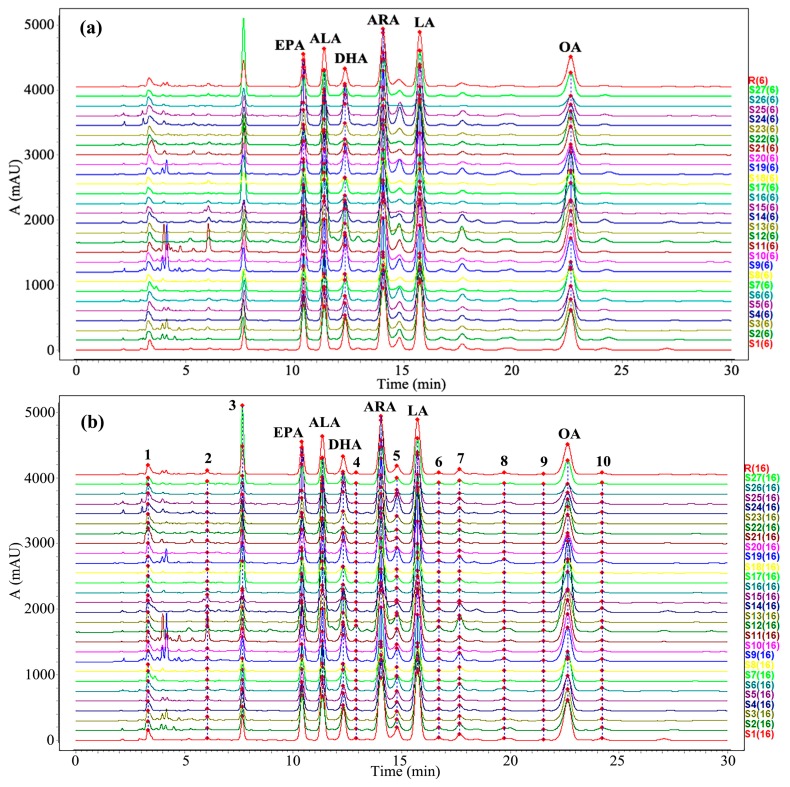
HPLC fingerprint chromatogram of 27 batches of *Oviductus Ranae*. (**a**) The fingerprint with six known PUFA peaks and (**b**) The fingerprint of 16 peaks with a peak area ratio greater than 0.5%.

In the first method, the fingerprint with six common peaks was obtained by matching the chromatographic peaks corresponding to the six kinds of PUFAs quantitatively analyzed, as shown in Figure 5a. The reference chromatogram R(6) of the fingerprint was created with the abbreviation of six kinds of PUFAs as the chromatographic peak number. The similarity coefficient between the chromatograms of 27 samples and reference chromatogram ranged from 0.913 to 0.995, showing a high degree of similarity. The reference chromatogram R(6) and the specific similarity data matrix are respectively listed in the supplementary data in Figure S1 and Table S2.

In the second method, by matching a common peak with area greater than 0.5%, the fingerprint with sixteen common peaks was obtained. The chromatographic peaks of the six kinds of PUFAs were also numbered by their abbreviations, and the rest chromatographic peaks were labeled with number 1–10 in chronological order (Figure 5b). The reference chromatogram R(16) of fingerprint was further created and showed in the supplementary data Figure S2. The similarity coefficient between chromatograms of samples and reference chromatogram ranged from 0.848 to 0.995, which was similar to the result of the method one. The specific similarity data matrix of method two is shown in the supplementary data in Table S3.

**Figure 6:**
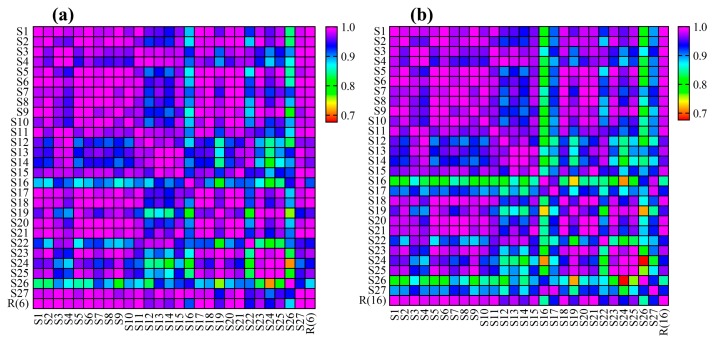
The heat maps of fingerprint similarity data matrices. (**a**) The heat map of method one and (**b**) The heat map of method two.

The similarity data matrices (Tables S2 and S3) obtained from the two methods were converted into heat maps to intuitively reveal the relationship and difference between the two fingerprint methods (Figure 6a,b). When comparing Figure 6a,b, the overall layout of the two heat maps was roughly the same, which reflected that the two fingerprint methods were generally consistent. This is because the contents of six PUFAs in petroleum ether extract were higher than the other components. Their peak areas accounted for a large proportion, occupying a dominant position in the calculation of the total peak area, so they played significant roles in the quality evaluation of *Oviductus Ranae*.

**Figure 7:**
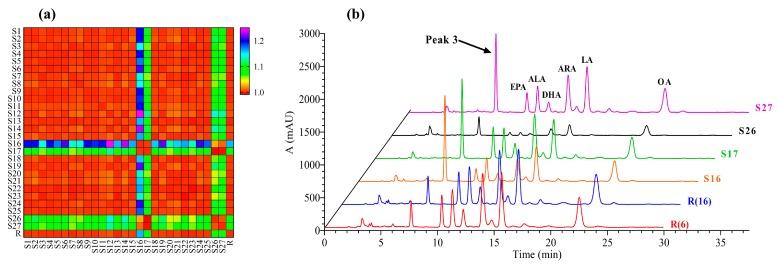
(**a**) The heat map showed the difference of similarity matrix between two fingerprint methods, (**b**) Comparison of samples chromatograms (S16, S17, S26, and S27) and reference chromatograms (R(6), R(16)).

A new data matrix (supplementary data Table S4) was created and converted to a heat map through dividing the similarity data matrix of the first method (Table S2) by the corresponding data in the matrix of the second method (Table S3) (Figure 7a). In Figure 7a, the similarity difference between the two methods was gradually increased from red to blue, and there were significant differences for the similarity of samples S16, S17, S26, and S27 in the two fingerprint methods. The chromatograms of samples S16, S17, S26, and S27 were compared with reference chromatogram R(6) and reference chromatogram R(16) (Figure 7b). The difference was found to be due to an increase in the common peak area of the common Peak 3. Therefore, reducing the proportion of the peak area of the six kinds of PUFAs in the total peak area will result in the absence of partial fingerprint information in the first method.

The fingerprint is usually a wide data matrix that is characterized by a large number of variables. Further analysis of multiple variables revealed that both fingerprint methods were feasible when the total peak area ratio of the six PUFAs in the sample was greater than 74.61%. The first method only matched the chromatographic peaks of six PUFAs, which facilitated the quantitative analysis of the known compounds, while the second method had more matching peaks and more accurately reflected the details of fingerprint.

### 3.4. Analysis of PUFAs in Oviductus Ranae

The six kinds of PUFAs that have been identified were selected for quantitative analysis in order to explore the quality differences of *Oviductus Ranae* samples from 27 various sampling sites. Table 4 shows the results of the determination.

In this study, the parameter P was applied to reflect the quality fluctuations between different batches of *Oviductus Ranae*. The closer the P value is to 100%, the better the consistency between different batches. Generally, the value of 75–125% was to be considered acceptable [48]. The formula for calculation was as follows:

$$P=\frac{C_{𝒾}}{{\bar{C}}_{𝒾}}\times 100\%$$

**Figure 8:**
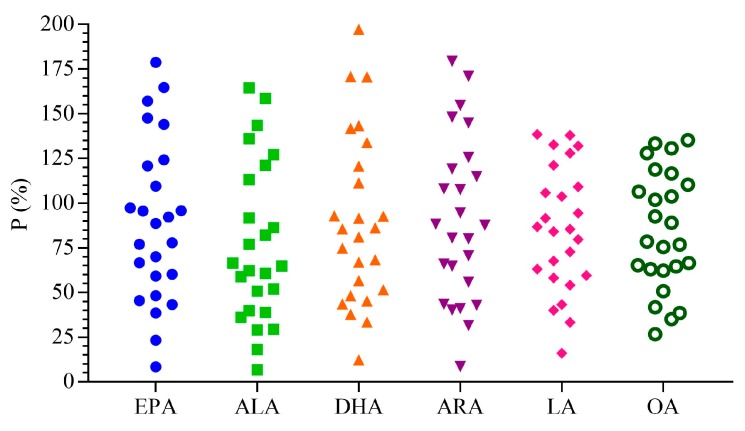
The difference of PUFAs contents in 27 batches of *Oviductus Ranae* samples.

Among them, $C_{𝒾}$ denoted the measured concentration of a certain component, while ${\bar{C}}_{𝒾}$ denoted its average concentration in 27 batches of *Oviductus Ranae*. As shown in Figure 8, the P values of the six kinds of PUFAs components varied greatly, far beyond the range of 75–125%, which indicated that the quality of different samples might fluctuate greatly.

### 3.5. Chemometric Analysis

#### 3.5.1. Hierarchical Cluster Analysis (HCA)

**Figure 9:**
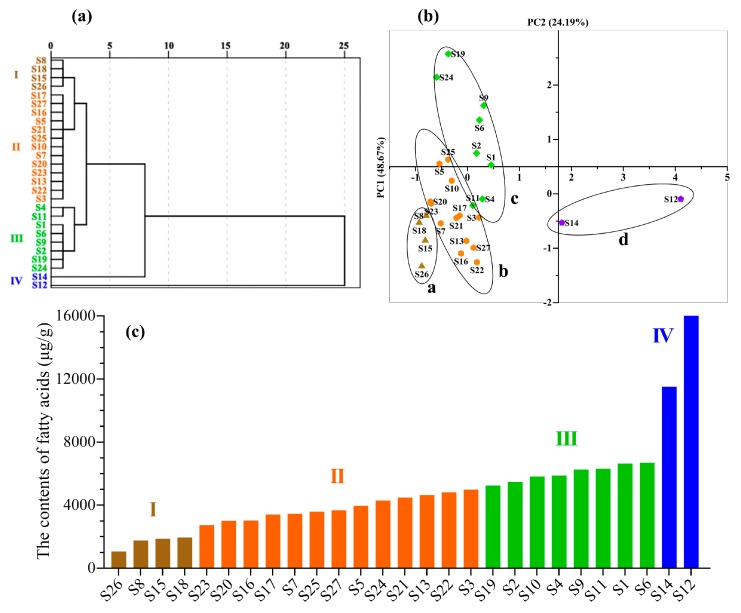
Unsupervised pattern recognition model of chemometrics. (**a**) The result of HCA of 27 batches of *Oviductus Ranae*, (**b**) PCA score chart of 27 batches of *Oviductus Ranae* in the first two principal components (PCs), and (**c**) The order of total content of six PUFAs in 27 batches of *Oviductus Ranae*.

HCA was used to classify *Oviductus Ranae* into different categories to further classify the *Oviductus Ranae* of different quality in Changbai Mountains and avoid subjective classification of human factors. As an unsupervised pattern recognition method, HCA can intuitively determine the similarity and difference of different samples from the tree diagram [40]. The HCA of the *Oviductus Ranae* samples was based on sixteen common peaks in the fingerprint, and the results are shown in Figure 9a. It was observed that the HCA could effectively distinguish the quality difference of *Oviductus Ranae* based on the total content of PUFAs when combined with the order of total content of six PUFAs in 27 batches of *Oviductus Ranae* (Figure 9c). Two samples with significantly higher PUFA contents (S12, S14) were classified in Cluster IV, and the four samples with the lowest PUFA contents (S8, S15, S18, and S26) were classified in Cluster I. Twenty-one samples with moderate PUFA contents were classified into two clusters, in which Cluster II contained thirteen samples and Cluster III contained eight samples. HCA successfully classified the excellent samples with high PUFA contents and the inferior samples with low PUFA contents in *Oviductus Ranae*. At the same time, twenty-one samples with medium content were divided into two categories, which achieved the effect of quality evaluation.

#### 3.5.2. Principal Component Analysis (PCA)

PCA has been used as an exploratory method to study sample classification for a long time. The PCA analysis was performed on the samples using SPSS, based on sixteen common peaks of the sample fingerprint in order to directly reflect the differences between 27 batches of *Oviductus Ranae* samples. Based on the eigenvalue > 1, the variance contribution rates of the first two principal components (PC1, PC2) were 48.67% (R^2^X[1]) and 24.19% (R^2^X[2]), respectively, and the cumulative variance contribution rate was 72.86%. This showed that the two principal components contain most information of all the variables, and the original data were fully reflected. The score chart was drawn while using the two principal components (PC1, PC2) to visualize the results (Figure 9b). Corresponding to the analysis results of HCA, the scores of 27 *Oviductus Ranae* samples can be divided into four regions in Figure 9b. Region a was four inferior samples with low PUFAs content, region b and region c with medium PUFAs content contained thirteen samples and eight samples, respectively, and region d was sample S12 and S14 with high PUFAs content. These results were consistent with the results of the HCA analysis, and different quality *Oviductus Ranae* samples were successfully classified.

#### 3.5.3. Partial Least Squares Discrimination Analysis (PLS-DA)

**Figure 10:**
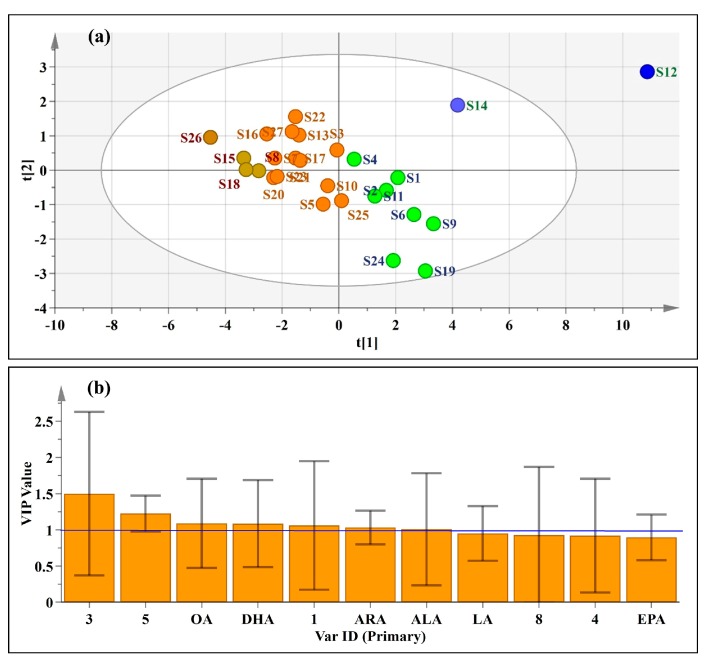
Supervised pattern recognition model. (**a**) partial least squares linear analysis (PLS-DA) score scatter plot of 27 batches of *Oviductus Ranae*, (**b**) The variable important for the project (VIP) plot of PLS-DA.

As a supervisory recognition model, PLS-DA can maximize the differences between different groups and help to filter the markers that are responsible for class separation. In the PLS-DA model, the 27 *Oviductus Ranae* samples were pre-classified into four categories according to the PCA results, and the scores are shown in Figure 10a. The PLS-DA model had satisfactory descriptiveness, but it relatively lacked predictability, as shown by the correlation coefficients (R^2^X(cum) = 0.786, R^2^Y(cum) = 0.591, Q^2^(cum) = 0.313). The PLS-DA scores showed consistent with the results of HCA and PCA. The four predetermined categories each presented a tightly distributed state, in which the high-quality samples S12 and S14 were separately divided, and the four inferior samples (S8, S15, S18, and S26) were divided on the leftmost side of Figure 10a.

The variable importance for the projection (VIP) diagram (Figure 10b) was constructed to determine the importance of each variable to the classification, which summarized the importance of variables for interpreting X and associating it with Y [49]. Chemical variables with a VIP value greater than 1 are considered to play an important role in the classification [41]. In this PLS-DA model, the VIP values of seven variables were greater than 1, including three unknown components (peak 3, peak 5, and peak 1) and four known PUFAs (OA, DHA, ARA, and ALA). In the variable of VIP > 1, some variables (excluding peak 5 and ARA) showed large error bars due to the limited number of samples in this work, however the fluctuation of error bars was within the range of the histogram, which is of statistical significance. It further indicated that the content of PUFAs played a significant role in the quality evaluation of *Oviductus Ranae*.

## 4. Conclusions

In this study, HPLC was used to analyze the petroleum ether extracts of 27 batches of *Oviductus Ranae* in the main producing areas of Changbai Mountains. Two types of chromatographic fingerprints were established with six common peaks of six high content of PUFAs and sixteen common chromatographic peaks with peak area > 0.5%, respectively, which could provide reference for the consistency evaluation of *Oviductus Ranae*. The chromatographic fingerprint that was established by matching six known PUFAs peaks was used for the quantitative. In order to further classify the *Oviductus Ranae*, two unsupervised pattern recognition models (PCA and HCA) and a supervised pattern recognition model (PLS-DA) were established by using the chemometric methods to analyze the fingerprint of sixteen chromatographic peaks with a peak area > 0.5%. All three chemometric models obtained consistent results. The *Oviductus Ranae* samples with different fatty acid content were successfully divided into four categories, among which the samples (S12, S14) with significant higher content were divided into one group and those (S8, S15, S18, and S26) with significantly lower content were clustered into one group. The results showed that the method has potential application value in the quality evaluation of *Oviductus Ranae*.

In conclusion, the study developed a feasible method for the evaluation of the quality of *Oviductus Ranae* while using HPLC fingerprint combined with chemometrics. It can provide reference for the establishment of quality criteria for regulatory authorities, increase people’s confidence in the effectiveness and safety of related nutritious products, and provide a reliable basis for the wide application of *Oviductus Ranae*.

## Figures and Tables

**Table 1:** Origin and collection date of *Oviductus Ranae* samples.

No.	Origin	Collection Date
S1	Jilin, Jilin	December 2016
S2	Jilin, Jilin	December 2016
S3	Jilin, Jilin	December 2016
S4	Jilin, Jilin	December 2016
S5	Jilin, Jilin	December 2016
S6	Jilin, Jilin	November 2015
S7	Jilin, Jilin	November 2015
S8	Jilin, Jilin	December 2016
S9	Baishan, Jilin	December 2016
S10	Baishan, Jilin	November 2015
S11	Baishan, Jilin	December 2016
S12	Baishan, Jilin	December 2016
S13	Baishan, Jilin	December 2016
S14	Baishan, Jilin	November 2015
S15	Baishan, Jilin	December 2016
S16	Baishan, Jilin	December 2016
S17	Baishan, Jilin	December 2016
S18	Tonghua, Jilin	December 2016
S19	Tonghua, Jilin	January 2016
S20	Tonghua, Jilin	December 2016
S21	Tonghua, Jilin	December 2016
S22	Tonghua, Jilin	December 2016
S23	Yanbian, Jilin	March 2016
S24	Yanbian, Jilin	March 2016
S25	Yanbian, Jilin	December 2016
S26	Yanbian, Jilin	December 2016
S27	Yanbian, Jilin	March 2016

**Table 2:** Regression equation of six kinds of PUFAs standards.

Compounds	Regression Equation	*R* ^2^	Linearity Range (μg/mL)
EPA	y = 11198x + 94.40	0.9999	14.59–116.74
ALA	y = 6244x + 91.95	0.9999	21.76–174.07
DHA	y = 14151x + 34.20	1.0000	7.20–57.57
ARA	y = 10379x + 505.69	0.9996	38.34–306.73
LA	y = 4221x + 522.27	0.9994	68.35–546.82
OA	y = 817x + 866.82	0.9982	303.56–2428.48

PUFA, polyunsaturated fatty acid; EPA, Eicosapentaenoic acid; ALA, α-linolenic acid; DHA, docosahexaenoic acid; ARA, arachidonic acid; LA, linoleic acid; OA, oleic acid.

**Table 3:** The relative standard deviation (RSD) results of methodology validation (*n* = 6).

Peak No.	Precision RSD		Repeatability RSD		Stability RSD	
	Retention Time	Peak Area	Retention Time	Peak Area	Retention Time	Peak Area
EPA	0.29%	1.90%	1.37%	0.73%	0.75%	0.64%
ALA	0.35%	2.16%	1.04%	2.18%	0.86%	0.74%
DHA	0.38%	3.01%	0.57%	3.62%	0.90%	0.92%
ARA	0.48%	2.90%	1.12%	3.28%	1.08%	0.85%
LA	0.51%	1.79%	1.03%	0.73%	1.15%	0.21%
OA	0.65%	1.91%	1.86%	1.51%	1.35%	0.25%

RSD, the relative standard deviation.

**Table 4:** The contents of PUFAs in 27 batches of *Oviductus Ranae* samples.

No	EPA (μg/g)	ALA (μg/g)	DHA (μg/g)	ARA (μg/g)	LA (μg/g)	OA (μg/g)	Total PUFAs (μg/g)
S1	214.09 ± 6.68	465.34 ± 19.06	115.22 ± 2.45	471.10 ± 10.03	1085.30 ± 33.89	4289.44 ± 91.30	6640.49 ± 207.35
S2	142.43 ± 5.83	253.48 ± 5.39	95.69 ± 3.31	460.82 ± 15.96	1120.31 ± 45.88	3408.23 ± 118.06	5480.96 ± 224.45
S3	124.53 ± 2.65	332.17 ± 11.51	57.82 ± 0.86	255.86 ± 3.79	725.47 ± 15.44	3494.53 ± 51.71	4990.39 ± 106.21
S4	115.29 ± 2.45	482.87 ± 16.73	62.64 ± 0.93	300.60 ± 4.45	1028.25 ± 21.88	3901.76 ± 57.74	5891.39 ± 125.39
S5	126.43 ± 2.69	152.58 ± 5.29	54.68 ± 0.81	342.29 ± 5.07	713.37 ± 15.18	2577.81 ± 38.15	3967.16 ± 84.44
S6	187.37 ± 6.49	373.22 ± 5.52	96.78 ± 3.44	491.98 ± 17.48	1169.85 ± 40.52	4380.19 ± 155.67	6699.38 ± 232.07
S7	86.57 ± 3.00	106.14 ± 1.57	45.18 ± 1.61	206.08 ± 7.32	493.37 ± 17.09	2524.24 ± 89.71	3461.58 ± 119.91
S8	59.09 ± 0.87	52.98 ± 1.88	29.38 ± 0.90	130.51 ± 4.00	340.31 ± 5.04	1153.14 ± 35.34	1765.41 ± 26.13
S9	232.37 ± 3.44	399.19 ± 14.19	133.14 ± 4.08	543.52 ± 16.66	1125.50 ± 16.66	3827.95 ± 117.30	6261.68 ± 92.66
S10	101.23 ± 1.50	148.88 ± 5.29	62.68 ± 1.92	399.61 ± 12.25	897.53 ± 13.28	4203.24 ± 128.80	5813.18 ± 86.03
S11	161.58 ± 5.74	421.28 ± 12.91	75.14 ± 2.13	343.60 ± 9.74	880.00 ± 31.27	4442.21 ± 125.96	6323.82 ± 224.74
S12	345.16 ± 12.27	1602.63 ± 49.11	143.55 ± 4.07	729.40 ± 20.68	2807.28 ± 99.77	11109.46 ± 315.01	16737.47 ± 594.83
S13	77.00 ± 2.74	240.83 ± 7.38	34.85 ± 0.99	177.32 ± 5.03	776.62 ± 27.60	3344.39 ± 94.83	4651.01 ± 165.29
S14	157.19 ± 4.82	702.42 ± 19.92	90.36 ± 2.11	378.98 ± 8.83	1958.06 ± 60.00	8232.10 ± 191.83	11519.11 ± 352.98
S15	30.26 ± 0.93	85.19 ± 2.42	22.63 ± 0.53	100.18 ± 2.33	367.33 ± 11.26	1266.76 ± 29.52	1872.34 ± 57.37
S16	50.02 ± 1.42	177.98 ± 4.15	32.49 ± 1.41	127.79 ± 5.53	505.00 ± 14.32	2145.41 ± 92.90	3038.70 ± 86.16
S17	124.29 ± 3.52	225.99 ± 5.27	58.18 ± 2.52	254.30 ± 11.01	573.60 ± 16.26	2186.20 ± 94.67	3422.57 ± 97.05
S18	56.38 ± 1.60	86.10 ± 2.01	30.58 ± 1.32	136.73 ± 5.92	282.63 ± 8.01	1371.83 ± 59.40	1964.25 ± 55.70
S19	204.33 ± 4.76	190.00 ± 8.23	115.27 ± 4.32	640.97 ± 24.04	1174.22 ± 27.36	2921.16 ± 109.57	5245.95 ± 122.24
S20	91.16 ± 2.12	114.00 ± 4.94	50.49 ± 1.89	224.45 ± 8.42	459.50 ± 10.71	2071.03 ± 77.68	3010.64 ± 70.16
S21	119.93 ± 2.79	182.93 ± 7.92	61.78 ± 2.32	278.98 ± 10.46	801.59 ± 18.68	3039.26 ± 114.00	4484.46 ± 104.50
S22	62.61 ± 2.71	355.43 ± 13.33	25.50 ± 0.47	138.36 ± 2.54	617.66 ± 26.75	3624.21 ± 66.43	4823.76 ± 208.87
S23	100.14 ± 4.34	117.11 ± 4.39	46.09 ± 0.84	280.98 ± 5.15	535.16 ± 23.17	1666.96 ± 30.56	2746.45 ± 118.92
S24	260.79 ± 9.78	269.35 ± 4.94	154.45 ± 4.82	570.49 ± 17.81	925.92 ± 34.73	2120.20 ± 66.20	4301.20 ± 161.34
S25	191.92 ± 7.20	172.66 ± 3.16	81.40 ± 2.54	365.47 ± 11.41	736.09 ± 27.61	2043.51 ± 63.81	3591.06 ± 134.70
S26	10.88 ± 0.20	19.81 ± 0.62	8.31 ± 0.34	27.07 ± 1.11	136.43 ± 2.50	878.52 ± 35.98	1081.01 ± 19.82
S27	78.25 ± 1.43	194.87 ± 6.08	38.27 ± 1.57	209.59 ± 8.58	676.80 ± 12.41	2478.02 ± 101.48	3675.80 ± 67.38

Each *Oviductus Ranae* sample was extracted three times in parallel and measured. The values are expressed as mean ± standard deviation of the PUFAs in each sample.

## Supplementary Materials

The following are available online at https://www.mdpi.com/2304-8158/8/8/322/s1, Figure S1: The reference chromatogram R(6) of the fingerprint with six common peaks, Figure S2: The reference chromatogram R(16) of the fingerprint with sixteen common peaks, Table S1: The contents of six kinds of PUFAs in S12, S22 and S26 of three different sampling weights, Table S2: The specific similarity data matrix of the fingerprint with six common peaks in the first method, Table S3: The specific similarity data matrix of the fingerprint with sixteen common peaks in the second method, Table S4: The difference of similarity data matrices between two fingerprint methods.
