# Supplementary material for: Quality Evaluation of Oviductus Ranae Based on PUFAs Using HPLC Fingerprint Techniques Combined with Chemometric Methods
Source: Foods. 2019 Aug 7;8(8):322. doi: 10.3390/foods8080322 (PMC6723783; doi:10.3390/foods8080322)
Supplement: Supplementary file 1 [file foods-08-00322-s001.pdf]

Supplementary Materials:

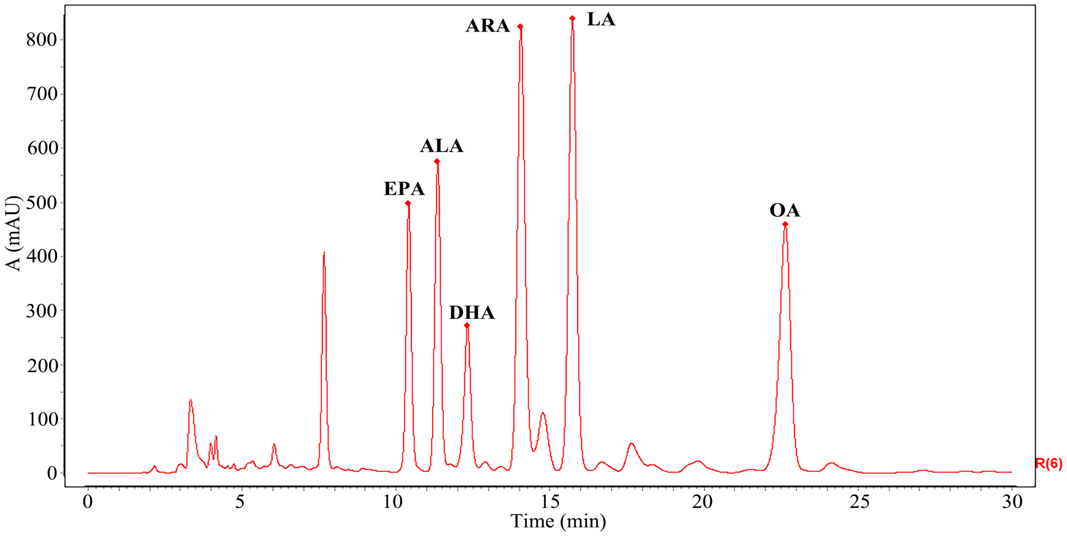

Figure 1. The reference chromatogram R(6) of the fingerprint with six common peaks.

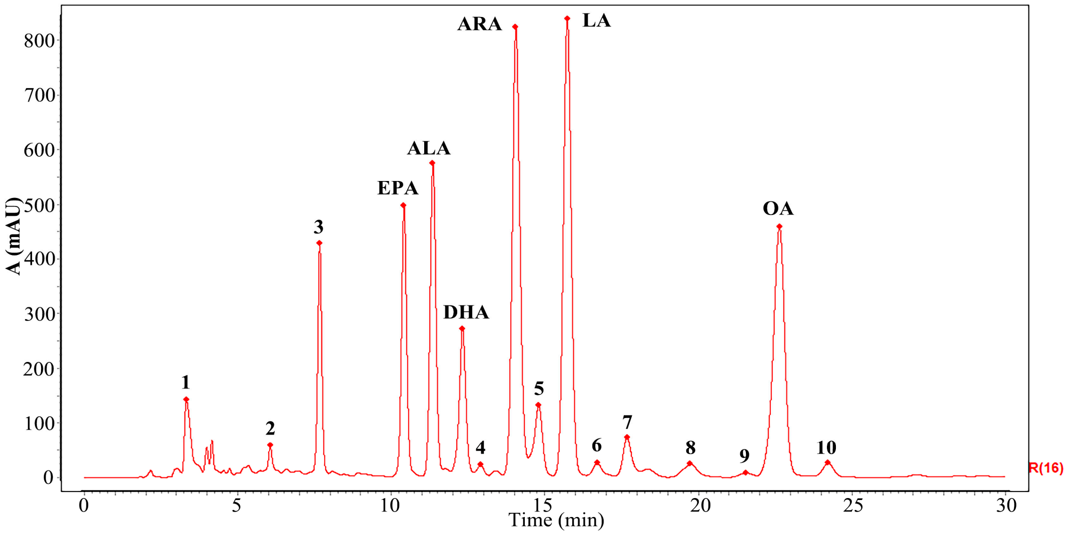

Figure 2. The reference chromatogram R(16) of the fingerprint with sixteen common peaks.

Table S1. The contents of PUFAs in S12, S22 and S26 of three different sampling weights.

Samples	Weight (g)	EPA (μg/g)	ALA (μg/g)	DHA (μg/g)	ARA (μg/g)	LA (μg/g)	OA (μg/g)	Total PUFAs (μg/g)
S26	0.80 [a]	10.88 ± 0.20	19.81 ± 0.62	8.31 ± 0.34	27.07 ± 1.11	136.43 ± 2.50	878.52 ± 35.98	1081.01 ± 19.82
	1.60 [a]	11.00 ± 0.49	19.48 ± 0.70	8.25 ± 0.42	28.74 ± 1.40	131.07 ± 6.39	875.84 ± 38.59	1074.38 ± 47.28
	3.20 [a]	10.55 ± 0.69	20.21 ± 1.66	8.01 ± 0.48	29.27 ± 1.52	136.99 ± 5.78	884.72 ± 55.75	1089.74 ± 62.90
	RSD [b]	4.42%	5.05%	4.72%	5.42%	3.93%	4.39%	3.80%
S22	0.80 [a]	62.61 ± 2.71	355.43 ± 13.33	25.50 ± 0.47	138.36 ± 2.54	617.66 ± 26.75	3624.21 ± 66.43	4823.76 ± 208.87
	1.60 [a]	60.89 ± 2.91	344.43 ± 11.94	24.62 ± 0.62	133.16 ± 2.96	607.52 ± 10.04	3531.14 ± 57.45	4701.77 ± 81.77
	3.20 [a]	62.08 ± 2.72	341.02 ± 14.77	25.46 ± 0.91	133.21 ± 2.82	622.59 ± 27.30	3462.64 ± 105.87	4647.01 ± 147.57
	RSD [b]	4.08%	3.84%	2.92%	2.62%	3.39%	2.78%	3.29%
S12	0.80 [a]	345.16 ± 12.27	1602.63 ± 49.11	143.55 ± 4.07	729.40 ± 20.68	2807.28 ± 99.77	11109.46 ± 315.01	16737.47 ± 594.83
	1.60 [a]	335.21 ± 4.05	1506.47 ± 73.13	137.30 ± 5.90	697.65 ± 29.97	2661.84 ± 158.01	10529.63 ± 405.67	15868.10 ± 655.08
	3.20 [a]	316.28 ± 18.59	1616.91 ± 109.68	138.77 ± 12.00	670.51 ± 42.77	2746.53 ± 97.31	11016.81 ± 502.86	16505.80 ± 723.54
	RSD [b]	5.12%	5.55%	5.39%	5.43%	4.49%	4.13%	4.23%

[a] Each *Oviductus Ranae* sample with different weight was measured three times in parallel, and the values are expressed as mean ± standard deviation of the PUFAs in each sample. [b] The relative standard deviations (RSD) of nine measurements of representative samples (three different weights, three parallel measurements of each weight).

Table S2. The specific similarity data matrix of the fingerprint with six common peaks in the first method.

No	S1	S2	S3	S4	S5	S6	S7	S8	S9	S10	S11	S12	S13	S14	S15	S16	S17	S18	S19	S20	S21	S22	S23	S24	S25	S26	S27	R(6)
S1	1.000	0.984	0.989	0.978	0.982	0.996	0.977	0.978	0.995	0.967	0.991	0.960	0.954	0.940	0.966	0.966	0.999	0.988	0.957	0.986	0.985	0.924	0.977	0.956	0.976	0.880	0.982	0.995
S2	0.984	1.000	0.964	0.957	0.995	0.995	0.978	0.993	0.990	0.985	0.965	0.923	0.946	0.925	0.977	0.955	0.986	0.984	0.984	0.989	0.991	0.883	0.993	0.962	0.982	0.880	0.983	0.989
S3	0.989	0.964	1.000	0.992	0.961	0.984	0.978	0.966	0.969	0.963	0.999	0.981	0.983	0.975	0.978	0.990	0.985	0.982	0.912	0.973	0.983	0.970	0.946	0.902	0.939	0.927	0.989	0.993
S4	0.978	0.957	0.992	1.000	0.944	0.974	0.955	0.949	0.956	0.945	0.989	0.994	0.984	0.984	0.978	0.991	0.972	0.959	0.897	0.949	0.972	0.973	0.929	0.882	0.919	0.907	0.987	0.986
S5	0.982	0.995	0.961	0.944	1.000	0.993	0.985	0.994	0.991	0.989	0.965	0.907	0.935	0.908	0.965	0.943	0.988	0.991	0.987	0.995	0.988	0.874	0.998	0.967	0.988	0.881	0.975	0.985
S6	0.996	0.995	0.984	0.974	0.993	1.000	0.986	0.991	0.994	0.984	0.986	0.948	0.960	0.943	0.979	0.969	0.997	0.993	0.970	0.993	0.994	0.917	0.988	0.956	0.980	0.896	0.990	0.998
S7	0.977	0.978	0.978	0.955	0.985	0.986	1.000	0.991	0.971	0.994	0.979	0.923	0.966	0.943	0.980	0.971	0.980	0.997	0.947	0.994	0.993	0.922	0.971	0.922	0.959	0.946	0.984	0.986
S8	0.978	0.993	0.966	0.949	0.994	0.991	0.991	1.000	0.982	0.990	0.966	0.913	0.954	0.930	0.980	0.959	0.983	0.990	0.972	0.994	0.996	0.889	0.987	0.949	0.980	0.912	0.984	0.987
S9	0.995	0.990	0.969	0.956	0.991	0.994	0.971	0.982	1.000	0.968	0.973	0.929	0.929	0.909	0.954	0.942	0.996	0.985	0.980	0.989	0.981	0.882	0.991	0.980	0.991	0.850	0.970	0.987
S10	0.967	0.985	0.963	0.945	0.989	0.984	0.994	0.990	0.968	1.000	0.965	0.907	0.955	0.930	0.979	0.960	0.971	0.991	0.960	0.991	0.989	0.901	0.978	0.923	0.956	0.933	0.980	0.981
S11	0.991	0.965	0.999	0.989	0.965	0.986	0.979	0.966	0.973	0.965	1.000	0.977	0.976	0.967	0.973	0.984	0.988	0.985	0.919	0.977	0.982	0.964	0.951	0.911	0.944	0.920	0.986	0.993
S12	0.960	0.923	0.981	0.994	0.907	0.948	0.923	0.913	0.929	0.907	0.977	1.000	0.972	0.979	0.953	0.978	0.951	0.929	0.852	0.916	0.944	0.979	0.890	0.845	0.884	0.883	0.966	0.965
S13	0.954	0.946	0.983	0.984	0.935	0.960	0.966	0.954	0.929	0.955	0.976	0.972	1.000	0.996	0.990	0.998	0.951	0.956	0.874	0.946	0.976	0.978	0.912	0.841	0.896	0.962	0.989	0.975
S14	0.940	0.925	0.975	0.984	0.908	0.943	0.943	0.930	0.909	0.930	0.967	0.979	0.996	1.000	0.980	0.995	0.934	0.933	0.843	0.920	0.958	0.984	0.883	0.811	0.868	0.949	0.978	0.962
S15	0.966	0.977	0.978	0.978	0.965	0.979	0.980	0.980	0.954	0.979	0.973	0.953	0.990	0.980	1.000	0.992	0.966	0.972	0.924	0.968	0.991	0.945	0.949	0.886	0.931	0.948	0.997	0.986
S16	0.966	0.955	0.990	0.991	0.943	0.969	0.971	0.959	0.942	0.960	0.984	0.978	0.998	0.995	0.992	1.000	0.962	0.965	0.887	0.955	0.980	0.978	0.923	0.859	0.908	0.954	0.992	0.983
S17	0.999	0.986	0.985	0.972	0.988	0.997	0.980	0.983	0.996	0.971	0.988	0.951	0.951	0.934	0.966	0.962	1.000	0.991	0.964	0.990	0.988	0.915	0.983	0.962	0.983	0.879	0.981	0.995
S18	0.988	0.984	0.982	0.959	0.991	0.993	0.997	0.990	0.985	0.991	0.985	0.929	0.956	0.933	0.972	0.965	0.991	1.000	0.960	0.998	0.991	0.917	0.982	0.944	0.971	0.920	0.982	0.991
S19	0.957	0.984	0.912	0.897	0.987	0.970	0.947	0.972	0.980	0.960	0.919	0.852	0.874	0.843	0.924	0.887	0.964	0.960	1.000	0.972	0.957	0.794	0.995	0.986	0.989	0.802	0.936	0.953
S20	0.986	0.989	0.973	0.949	0.995	0.993	0.994	0.994	0.989	0.991	0.977	0.916	0.946	0.920	0.968	0.955	0.990	0.998	0.972	1.000	0.991	0.896	0.989	0.957	0.981	0.906	0.978	0.988
S21	0.985	0.991	0.983	0.972	0.988	0.994	0.993	0.996	0.981	0.989	0.982	0.944	0.976	0.958	0.991	0.980	0.988	0.991	0.957	0.991	1.000	0.925	0.977	0.933	0.969	0.928	0.995	0.995
S22	0.924	0.883	0.970	0.973	0.874	0.917	0.922	0.889	0.882	0.901	0.964	0.979	0.978	0.984	0.945	0.978	0.915	0.917	0.794	0.896	0.925	1.000	0.846	0.772	0.829	0.939	0.949	0.940
S23	0.977	0.993	0.946	0.929	0.998	0.988	0.971	0.987	0.991	0.978	0.951	0.890	0.912	0.883	0.949	0.923	0.983	0.982	0.995	0.989	0.977	0.846	1.000	0.980	0.992	0.847	0.962	0.975
S24	0.956	0.962	0.902	0.882	0.967	0.956	0.922	0.949	0.980	0.923	0.911	0.845	0.841	0.811	0.886	0.859	0.962	0.944	0.986	0.957	0.933	0.772	0.980	1.000	0.990	0.749	0.908	0.937
S25	0.976	0.982	0.939	0.919	0.988	0.980	0.959	0.980	0.991	0.956	0.944	0.884	0.896	0.868	0.931	0.908	0.983	0.971	0.989	0.981	0.969	0.829	0.992	0.990	1.000	0.819	0.948	0.968
S26	0.880	0.880	0.927	0.907	0.881	0.896	0.946	0.912	0.850	0.933	0.920	0.883	0.962	0.949	0.948	0.954	0.879	0.920	0.802	0.906	0.928	0.939	0.847	0.749	0.819	1.000	0.934	0.913
S27	0.982	0.983	0.989	0.987	0.975	0.990	0.984	0.984	0.970	0.980	0.986	0.966	0.989	0.978	0.997	0.992	0.981	0.982	0.936	0.978	0.995	0.949	0.962	0.908	0.948	0.934	1.000	0.995
R(6)	0.995	0.989	0.993	0.986	0.985	0.998	0.986	0.987	0.987	0.981	0.993	0.965	0.975	0.962	0.986	0.983	0.995	0.991	0.953	0.988	0.995	0.940	0.975	0.937	0.968	0.913	0.995	1.000

Table S3. The specific similarity data matrix of the fingerprint with sixteen common peaks in the second method.

No	S1	S2	S3	S4	S5	S6	S7	S8	S9	S10	S11	S12	S13	S14	S15	S16	S17	S18	S19	S20	S21	S22	S23	S24	S25	S26	S27	R(16)
S1	1.000	0.983	0.985	0.978	0.981	0.995	0.973	0.971	0.994	0.967	0.986	0.958	0.953	0.939	0.961	0.805	0.911	0.984	0.956	0.980	0.978	0.924	0.974	0.956	0.975	0.799	0.895	0.994
S2	0.983	1.000	0.960	0.956	0.991	0.993	0.973	0.983	0.989	0.983	0.960	0.922	0.943	0.924	0.967	0.798	0.902	0.976	0.982	0.980	0.977	0.883	0.986	0.961	0.978	0.791	0.898	0.986
S3	0.985	0.960	1.000	0.989	0.961	0.980	0.979	0.964	0.966	0.962	0.993	0.971	0.983	0.967	0.975	0.864	0.929	0.981	0.908	0.973	0.979	0.970	0.948	0.899	0.939	0.870	0.931	0.992
S4	0.978	0.956	0.989	1.000	0.944	0.975	0.952	0.945	0.957	0.946	0.984	0.992	0.983	0.982	0.974	0.827	0.891	0.957	0.897	0.947	0.966	0.972	0.927	0.882	0.919	0.826	0.902	0.985
S5	0.981	0.991	0.961	0.944	1.000	0.992	0.982	0.991	0.990	0.988	0.961	0.903	0.935	0.904	0.964	0.804	0.913	0.990	0.985	0.993	0.984	0.876	0.996	0.965	0.987	0.816	0.901	0.984
S6	0.995	0.993	0.980	0.975	0.992	1.000	0.980	0.984	0.994	0.983	0.980	0.947	0.958	0.941	0.973	0.801	0.905	0.989	0.970	0.987	0.986	0.915	0.982	0.955	0.977	0.809	0.897	0.995
S7	0.973	0.973	0.979	0.952	0.982	0.980	1.000	0.988	0.967	0.991	0.973	0.914	0.967	0.935	0.978	0.854	0.927	0.995	0.940	0.994	0.990	0.924	0.972	0.917	0.956	0.891	0.929	0.986
S8	0.971	0.983	0.964	0.945	0.991	0.984	0.988	1.000	0.977	0.984	0.958	0.901	0.953	0.918	0.977	0.838	0.924	0.988	0.964	0.992	0.993	0.889	0.984	0.940	0.976	0.862	0.923	0.984
S9	0.994	0.989	0.966	0.957	0.990	0.994	0.967	0.977	1.000	0.968	0.969	0.927	0.927	0.908	0.949	0.785	0.908	0.981	0.980	0.983	0.973	0.882	0.986	0.979	0.989	0.772	0.883	0.986
S10	0.967	0.983	0.962	0.946	0.988	0.983	0.991	0.984	0.968	1.000	0.961	0.905	0.955	0.928	0.976	0.808	0.893	0.988	0.959	0.987	0.983	0.902	0.976	0.921	0.953	0.851	0.899	0.980
S11	0.986	0.960	0.993	0.984	0.961	0.980	0.973	0.958	0.969	0.961	1.000	0.968	0.971	0.960	0.968	0.824	0.904	0.979	0.914	0.967	0.971	0.959	0.946	0.907	0.941	0.836	0.901	0.988
S12	0.958	0.922	0.971	0.992	0.903	0.947	0.914	0.901	0.927	0.905	0.968	1.000	0.965	0.979	0.942	0.783	0.847	0.920	0.852	0.904	0.930	0.973	0.881	0.844	0.880	0.777	0.858	0.958
S13	0.953	0.943	0.983	0.983	0.935	0.958	0.967	0.953	0.927	0.955	0.971	0.965	1.000	0.990	0.988	0.861	0.893	0.957	0.872	0.946	0.973	0.978	0.915	0.840	0.897	0.895	0.924	0.976
S14	0.939	0.924	0.967	0.982	0.904	0.941	0.935	0.918	0.908	0.928	0.960	0.979	0.990	1.000	0.970	0.809	0.842	0.925	0.842	0.910	0.945	0.980	0.876	0.810	0.864	0.840	0.878	0.957
S15	0.961	0.967	0.975	0.974	0.964	0.973	0.978	0.977	0.949	0.976	0.968	0.942	0.988	0.970	1.000	0.854	0.902	0.973	0.916	0.967	0.990	0.944	0.948	0.879	0.928	0.891	0.927	0.983
S16	0.805	0.798	0.864	0.827	0.804	0.801	0.854	0.838	0.785	0.808	0.824	0.783	0.861	0.809	0.854	1.000	0.960	0.835	0.731	0.843	0.849	0.841	0.814	0.712	0.771	0.930	0.981	0.848
S17	0.911	0.902	0.929	0.891	0.913	0.905	0.927	0.924	0.908	0.893	0.904	0.847	0.893	0.842	0.902	0.960	1.000	0.925	0.871	0.936	0.923	0.859	0.926	0.870	0.904	0.887	0.986	0.930
S18	0.984	0.976	0.981	0.957	0.990	0.989	0.995	0.988	0.981	0.988	0.979	0.920	0.957	0.925	0.973	0.835	0.925	1.000	0.954	0.998	0.991	0.918	0.981	0.938	0.969	0.867	0.916	0.989
S19	0.956	0.982	0.908	0.897	0.985	0.970	0.940	0.964	0.980	0.959	0.914	0.852	0.872	0.842	0.916	0.731	0.871	0.954	1.000	0.964	0.945	0.793	0.987	0.985	0.985	0.718	0.845	0.950
S20	0.980	0.980	0.973	0.947	0.993	0.987	0.994	0.992	0.983	0.987	0.967	0.904	0.946	0.910	0.967	0.843	0.936	0.998	0.964	1.000	0.990	0.897	0.989	0.949	0.977	0.863	0.924	0.987
S21	0.978	0.977	0.979	0.966	0.984	0.986	0.990	0.993	0.973	0.983	0.971	0.930	0.973	0.945	0.990	0.849	0.923	0.991	0.945	0.990	1.000	0.924	0.976	0.922	0.963	0.883	0.928	0.991
S22	0.924	0.883	0.970	0.972	0.876	0.915	0.924	0.889	0.882	0.902	0.959	0.973	0.978	0.980	0.944	0.841	0.859	0.918	0.793	0.897	0.924	1.000	0.850	0.772	0.830	0.872	0.887	0.941
S23	0.974	0.986	0.948	0.927	0.996	0.982	0.972	0.984	0.986	0.976	0.946	0.881	0.915	0.876	0.948	0.814	0.926	0.981	0.987	0.989	0.976	0.850	1.000	0.973	0.990	0.808	0.907	0.975
S24	0.956	0.961	0.899	0.882	0.965	0.955	0.917	0.940	0.979	0.921	0.907	0.844	0.840	0.810	0.879	0.712	0.870	0.938	0.985	0.949	0.922	0.772	0.973	1.000	0.989	0.676	0.823	0.935
S25	0.975	0.978	0.939	0.919	0.987	0.977	0.956	0.976	0.989	0.953	0.941	0.880	0.897	0.864	0.928	0.771	0.904	0.969	0.985	0.977	0.963	0.830	0.990	0.989	1.000	0.760	0.874	0.966
S26	0.799	0.791	0.870	0.826	0.816	0.809	0.891	0.862	0.772	0.851	0.836	0.777	0.895	0.840	0.891	0.930	0.887	0.867	0.718	0.863	0.883	0.872	0.808	0.676	0.760	1.000	0.928	0.849
S27	0.895	0.898	0.931	0.902	0.901	0.897	0.929	0.923	0.883	0.899	0.901	0.858	0.924	0.878	0.927	0.981	0.986	0.916	0.845	0.924	0.928	0.887	0.907	0.823	0.874	0.928	1.000	0.929
R(16)	0.994	0.986	0.992	0.985	0.984	0.995	0.986	0.984	0.986	0.980	0.988	0.958	0.976	0.957	0.983	0.848	0.930	0.989	0.950	0.987	0.991	0.941	0.975	0.935	0.966	0.849	0.929	1.000

Table S4. The difference of similarity data matrices between two fingerprint methods.

No	S1	S2	S3	S4	S5	S6	S7	S8	S9	S10	S11	S12	S13	S14	S15	S16	S17	S18	S19	S20	S21	S22	S23	S24	S25	S26	S27	R
S1	1.000	1.001	1.004	1.000	1.001	1.001	1.004	1.007	1.001	1.000	1.005	1.002	1.001	1.001	1.005	1.200	1.097	1.004	1.001	1.006	1.007	1.000	1.003	1.000	1.001	1.101	1.097	1.001
S2	1.001	1.000	1.004	1.001	1.004	1.002	1.005	1.010	1.001	1.002	1.005	1.001	1.003	1.001	1.010	1.197	1.093	1.008	1.002	1.009	1.014	1.000	1.007	1.001	1.004	1.113	1.095	1.003
S3	1.004	1.004	1.000	1.003	1.000	1.004	0.999	1.002	1.003	1.001	1.006	1.010	1.000	1.008	1.003	1.146	1.060	1.001	1.004	1.000	1.004	1.000	0.998	1.003	1.000	1.066	1.062	1.001
S4	1.000	1.001	1.003	1.000	1.000	0.999	1.003	1.004	0.999	0.999	1.005	1.002	1.001	1.002	1.004	1.198	1.091	1.002	1.000	1.002	1.006	1.001	1.002	1.000	1.000	1.098	1.094	1.001
S5	1.001	1.004	1.000	1.000	1.000	1.001	1.003	1.003	1.001	1.001	1.004	1.004	1.000	1.004	1.001	1.173	1.082	1.001	1.002	1.002	1.004	0.998	1.002	1.002	1.001	1.080	1.082	1.001
S6	1.001	1.002	1.004	0.999	1.001	1.000	1.006	1.007	1.000	1.001	1.006	1.001	1.002	1.002	1.006	1.210	1.102	1.004	1.000	1.006	1.008	1.002	1.006	1.001	1.003	1.108	1.104	1.003
S7	1.004	1.005	0.999	1.003	1.003	1.006	1.000	1.003	1.004	1.003	1.006	1.010	0.999	1.009	1.002	1.137	1.057	1.002	1.007	1.000	1.003	0.998	0.999	1.005	1.003	1.062	1.059	1.000
S8	1.007	1.010	1.002	1.004	1.003	1.007	1.003	1.000	1.005	1.006	1.008	1.013	1.001	1.013	1.003	1.144	1.064	1.002	1.008	1.002	1.003	1.000	1.003	1.010	1.004	1.058	1.066	1.003
S9	1.001	1.001	1.003	0.999	1.001	1.000	1.004	1.005	1.000	1.000	1.004	1.002	1.002	1.001	1.005	1.200	1.097	1.004	1.000	1.006	1.008	1.000	1.005	1.001	1.002	1.101	1.099	1.001
S10	1.000	1.002	1.001	0.999	1.001	1.001	1.003	1.006	1.000	1.000	1.004	1.002	1.000	1.002	1.003	1.188	1.087	1.003	1.001	1.004	1.006	0.999	1.002	1.002	1.003	1.096	1.090	1.001
S11	1.005	1.005	1.006	1.005	1.004	1.006	1.006	1.008	1.004	1.004	1.000	1.009	1.005	1.007	1.005	1.194	1.093	1.006	1.005	1.010	1.011	1.005	1.005	1.004	1.003	1.100	1.094	1.005
S12	1.002	1.001	1.010	1.002	1.004	1.001	1.010	1.013	1.002	1.002	1.009	1.000	1.007	1.000	1.012	1.249	1.123	1.010	1.000	1.013	1.015	1.006	1.010	1.001	1.005	1.136	1.126	1.007
S13	1.001	1.003	1.000	1.001	1.000	1.002	0.999	1.001	1.002	1.000	1.005	1.007	1.000	1.006	1.002	1.159	1.065	0.999	1.002	1.000	1.003	1.000	0.997	1.001	0.999	1.075	1.070	0.999
S14	1.001	1.001	1.008	1.002	1.004	1.002	1.009	1.013	1.001	1.002	1.007	1.000	1.006	1.000	1.010	1.230	1.109	1.009	1.001	1.011	1.014	1.004	1.008	1.001	1.005	1.130	1.114	1.005
S15	1.005	1.010	1.003	1.004	1.001	1.006	1.002	1.003	1.005	1.003	1.005	1.012	1.002	1.010	1.000	1.162	1.071	0.999	1.009	1.001	1.001	1.001	1.001	1.008	1.003	1.064	1.076	1.003
S16	1.200	1.197	1.146	1.198	1.173	1.210	1.137	1.144	1.200	1.188	1.194	1.249	1.159	1.230	1.162	1.000	1.002	1.156	1.213	1.133	1.154	1.163	1.134	1.206	1.178	1.026	1.011	1.159
S17	1.097	1.093	1.060	1.091	1.082	1.102	1.057	1.064	1.097	1.087	1.093	1.123	1.065	1.109	1.071	1.002	1.000	1.071	1.107	1.058	1.070	1.065	1.062	1.106	1.087	0.991	0.995	1.070
S18	1.004	1.008	1.001	1.002	1.001	1.004	1.002	1.002	1.004	1.003	1.006	1.010	0.999	1.009	0.999	1.156	1.071	1.000	1.006	1.000	1.000	0.999	1.001	1.006	1.002	1.061	1.072	1.002
S19	1.001	1.002	1.004	1.000	1.002	1.000	1.007	1.008	1.000	1.001	1.005	1.000	1.002	1.001	1.009	1.213	1.107	1.006	1.000	1.008	1.013	1.001	1.008	1.001	1.004	1.117	1.108	1.003
S20	1.006	1.009	1.000	1.002	1.002	1.006	1.000	1.002	1.006	1.004	1.010	1.013	1.000	1.011	1.001	1.133	1.058	1.000	1.008	1.000	1.001	0.999	1.000	1.008	1.004	1.050	1.058	1.001
S21	1.007	1.014	1.004	1.006	1.004	1.008	1.003	1.003	1.008	1.006	1.011	1.015	1.003	1.014	1.001	1.154	1.070	1.000	1.013	1.001	1.000	1.001	1.001	1.012	1.006	1.051	1.072	1.004
S22	1.000	1.000	1.000	1.001	0.998	1.002	0.998	1.000	1.000	0.999	1.005	1.006	1.000	1.004	1.001	1.163	1.065	0.999	1.001	0.999	1.001	1.000	0.995	1.000	0.999	1.077	1.070	0.999
S23	1.003	1.007	0.998	1.002	1.002	1.006	0.999	1.003	1.005	1.002	1.005	1.010	0.997	1.008	1.001	1.134	1.062	1.001	1.008	1.000	1.001	0.995	1.000	1.007	1.002	1.048	1.061	1.000
S24	1.000	1.001	1.003	1.000	1.002	1.001	1.005	1.010	1.001	1.002	1.004	1.001	1.001	1.001	1.008	1.206	1.106	1.006	1.001	1.008	1.012	1.000	1.007	1.000	1.001	1.108	1.103	1.002
S25	1.001	1.004	1.000	1.000	1.001	1.003	1.003	1.004	1.002	1.003	1.003	1.005	0.999	1.005	1.003	1.178	1.087	1.002	1.004	1.004	1.006	0.999	1.002	1.001	1.000	1.078	1.085	1.002
S26	1.101	1.113	1.066	1.098	1.080	1.108	1.062	1.058	1.101	1.096	1.100	1.136	1.075	1.130	1.064	1.026	0.991	1.061	1.117	1.050	1.051	1.077	1.048	1.108	1.078	1.000	1.006	1.075
S27	1.097	1.095	1.062	1.094	1.082	1.104	1.059	1.066	1.099	1.090	1.094	1.126	1.070	1.114	1.076	1.011	0.995	1.072	1.108	1.058	1.072	1.070	1.061	1.103	1.085	1.006	1.000	1.071
R	1.001	1.003	1.001	1.001	1.001	1.003	1.000	1.003	1.001	1.001	1.005	1.007	0.999	1.005	1.003	1.159	1.070	1.002	1.003	1.001	1.004	0.999	1.000	1.002	1.002	1.075	1.071	1.000
